# Historical Moral Responsibility and Manipulation via Deletion

**DOI:** 10.1007/s10670-021-00409-6

**Published:** 2021-05-06

**Authors:** Gabriel De Marco

**Affiliations:** Uehiro Centre for Practical Ethics, University of Oxford, Oxford, Oxfordshire UK

## Abstract

In discussions on moral responsibility for actions, a commonly discussed case is one in which an agent is manipulated into performing some action. On some views, such agents lack responsibility for those actions partly because they issue from attitudes that were acquired in an inappropriate way. In this paper, it is argued that such views are in need of revision. After introducing a new problematic case of a manipulated agent, revisions are offered for specific views. The paper concludes with a discussion of the views in a broader context, as well as some potential implications of the revisions.

## Introduction

Consider a pair of cases presented by Mele (2006, pp. 168–169, 2019, pp. 64–65). In the past, Pat occasionally felt guilty about being a mediocre father and decided to change this. In order to do so, Pat designed and executed a long-term plan for self-improvement. After years of work, he ended up being the wonderful father that he is today. This process resulted in parental values that are such that it would take Pat a significant amount of time to change them.^1^ Further, they are so strong that, although his faculties for rational control are not impaired, he cannot do otherwise than make certain sacrifices for his children, because he can see so clearly what the situation is. Today, he decided to make a sacrifice and took out a large loan to pay for his daughter’s first year at an expensive liberal arts college. Pat, we can stipulate, autonomously possesses these parental values. When he decides to take out the loan to help his daughter, Pat is morally responsible for the decision.

Now compare Pat to Paul, a mediocre father who has, for many years, reflectively identified with his selfish values. A team of scientists determined what makes Pat such a great father and used that knowledge to make Paul more like Pat. As Paul slept, the scientists implanted Pat’s hierarchy of values in Paul and erased competing values. Now Paul is very much like Pat, and were he to critically reflect on his own values and priorities, he would conclude the same thing that Pat would of himself. When Paul awakes, he remembers his daughter’s wish to go to an exclusive liberal arts college and experiences a strong desire to take out a loan to help her do that. Paul is surprised by this and wonders why, all of a sudden, he cares so much about his daughter’s welfare and not very much about the new car he wanted. He figures that he has become tired of his selfish ways and he finally sees the importance of a father-daughter relationship, and “[w]hen he carefully reflects on his values, Paul…wholeheartedly embraces the idea of living such a life and the values that support it” (Mele, 2019, p. 65). Later that day, Paul decides to take out a loan to finance his daughter’s first year in college and does so. Due to his new parental values, he could not have done otherwise, in the same sense that Pat could not have done otherwise.

Although Pat and Paul are identical with respect to many responsibility-relevant features, many will judge that only Pat is morally responsible for his decision; Paul does not deserve credit for his decision and is not morally responsible for it. Supposing that this judgment is true, how can we account for this difference between Pat and Paul?^2^ Given the similarities between Pat and Paul at, and just before, the time of decision, various philosophers have produced *historical views* of moral responsibility to account for the difference. On such views, whether an agent is morally responsible for an action is partly determined by the history of the agent or the agent’s attitudes issuing in the action.

On one type of historical view, the relevant difference between agents like Pat and Paul is, in part, that unlike Pat, the attitudes leading to Paul’s decision were acquired in a way that bypassed his capacities for control over his mental life (Fischer, 2012, Chapter 11; Haji & Cuypers, 2008; McKenna, 2016; Mele, 2006, 2019). Call such views *bypassing views*.^3^ The relevant capacities are, for instance, the capacity to critically assess, endorse, and sustain one’s values (Fischer, 2012, p. 198; Haji & Cuypers, 2008, p. 30; McKenna, 2016, p. 97; Mele, 1995, pp. 118–120). From now on, I simply refer to the process of bypassing these capacities as “bypassing.”

In this paper, I argue that bypassing views are in need of revision. The debate about manipulated agents has focused on cases like that of Paul, in which the manipulator implants new attitudes via bypassing; and bypassing views have focused on the acquisition of the relevant attitudes. However, there are other ways of manipulating agents which can lead to similar results. I begin by presenting the case of Matt, in which an agent undergoes a similar change at the hands of manipulators, yet has no new implanted attitudes. His action does not issue from attitudes that were acquired via bypassing. I then offer an initial diagnosis of a relevant difference between agents like Matt and Paul, on the one hand, and typical, unmanipulated agents like Pat, on the other. With this diagnosis in hand, I discuss some bypassing views in detail, explaining how they accommodate traditional cases, and suggesting revisions that can help account for this new case. After offering these revisions, I compare the views in a broader context, and suggest some considerations that place limits on the revised views, and that will be relevant when further developing them.

## The Case of Matt

Consider Matt, whose history is similar to Pat’s, the unmanipulated good father above. When he was younger, Matt also wanted to avoid being a mediocre father. He decided to implement a project of self-improvement and succeeded. Matt still has some of his selfish values, and often makes decisions on the basis of these values. However, when it comes to choices concerning his daughter, she comes first, and his parental values win out. As a result of undergoing this program of self-improvement, Matt is now as good a father as Pat.

Now we can introduce the manipulators. A team of scientists wants to prevent Matt from taking out a loan for his daughter’s college education, and to make him take out a loan for a new car instead (the sort of thing that pre-manipulation Paul would have done). But they also want to be economical about it, and recognize that achieving this goal only requires that they remove Matt’s parental values. So, while Matt is sleeping, the scientists erase these values, and leave everything else intact. Since the scientists only erased his parental values, Matt is still capable of living a meaningful life, for he retains a plethora of other values. When Matt awakes, he recalls his desire to buy a new car, and he experiences a desire to make that possible by taking out a loan for the car. He decides to do this, instead of taking out a loan to fund his daughter’s education.

It seems that Matt is no more responsible for his decision than Paul was for his. Motivations sometimes offered for the claim that Paul is not responsible apply to Matt as well. Matt was not aware of the neuroscientists’ process, did not consent to it, and did not have the opportunity to resist it (Mele, 2006, p. 169). Further, by erasing his parental values,

the brainwashers gave his life a new direction that clashes with the considered principles and values he had before he was manipulated. He seems heteronomous – and unfree – to a significant extent, and he seems to…lack moral responsibility for [taking out the loan]. (Mele, 2006, p. 169).

A further motivation can be presented for the judgment about Matt which may not apply to Paul: the very part of his set of values that was erased is a part that Matt had worked hard on, for a significant amount of time, to produce; and he had done this work precisely in order to counteract some of his selfish attitudes in cases where they led him astray as a parent. As McKenna says of a different case, Matt “was robbed of a character in which [he] was deeply invested, and [his] moral responsibility for it was the product of [his] psychic labors” (2016, pp. 95–96).

However, the explanation that bypassing views offer for Paul’s lack of responsibility, which partly appeals to the fact that the decision issues from attitudes that were *acquired* by means of bypassing, does not apply to Matt’s decision. Although Matt had some values erased, no new ones were produced or acquired. Bypassing views are in need of revision if they are to account for the case of Matt.

Before getting into the details of these views, and the suggested revisions, it will be helpful to find what it is that Matt and Paul have in common. We can begin with the observation that these agents underwent changes in their attitudes by means of bypassing. Yet this is not precise enough. Suppose that, on his way to get the loan for his daughter’s education, Paul decides to obnoxiously honk repeatedly at the car in front of him. Surely he can still be responsible for this; the changes produced by the manipulators have nothing to do with this action, or how it was produced. This sort of case shows the problem of *benign manipulation*, manipulation that is not relevant to the action in question. The attitudes leading to Paul’s obnoxious honking do not seem to have a problematic history. The attitudes leading to his decision to take out the loan, on the other hand, were changed or acquired as a result of bypassing.

Singling out changes in the attitudes leading to action that were a result of bypassing helps to account for Paul’s decision to take out the loan while avoiding the problem of benign manipulation, but it still does not get the right verdict in the case of Matt. The attitudes issuing in his decision to take out a loan for a new car were the same ones he had before the manipulation, and with the same strength. The relevant change in the case of Matt is in the *relative* strength of his selfish attitudes, relative to competing attitudes.

However, attitudes that compete in one context of practical deliberation might not compete in another context. Suppose, for instance, that at some point prior to the manipulation, Matt deliberated about whether he should politely engage in conversation with his conspiracy theorist neighbor or whether he should avoid him and go to the beach with his daughter. In this context of deliberation, Matt’s selfish values and parental values recommend the same course of action; they do not compete. So, a more refined explanation would focus on the strength of the attitudes leading to action relative to attitudes that would compete *in this context of practical deliberation*, were he to still have them. When he was deliberating about whether to take out the loan for the car, his parental values would have competed with his selfish values. Further, since Matt lost his parental values, they are absent from this deliberation. In this case, as well as the case of Paul, the relevant competing attitudes are ones that the agent had prior to the change and would compete in this context of deliberation.^4^

Bringing all of this together, we can now identify a feature that Matt’s decision has in common with victims in standard manipulation cases, like Paul. Paul’s and Matt’s decisions issued from attitudes that, in virtue of bypassing, have a new relative strength. The relative strength of concern is in relation to attitudes had by the agent prior to the bypassing that would have competed, in this context of practical deliberation, with the attitudes that led to action. For ease of presentation, we can call such attitudes *B-attitudes*. One important feature of Matt’s and Paul’s decisions (which is absent in Pat’s decision) is that they issue from B-attitudes. In Paul’s case, his parental values were implanted via bypassing, and have a new relative strength; the parental values were B-attitudes. In the case of Matt, the change occurred via the elimination of his parental values, resulting in selfish values that are B-attitudes in this context of deliberation. This difference between Paul’s and Matt’s decisions, on the one hand, and Pat’s decision, on the other, is an important difference; but, as we will see later, we will need more to the story in order to avoid other concerns.

Before going further, we should consider an objection to my claim that traditional bypassing views cannot accommodate the verdict that Matt is not responsible. An important part of this case is that none of the attitudes that lead to his action were acquired through bypassing; this feature is necessary for the case to pose a problem for bypassing views. Yet, one might argue that the process of erasing Matt’s parental values *did* produce a new attitude, an attitude of indifference towards his daughter. It is not clear how this objection would best be developed, but doing so would likely involve various complications. In order to avoid this, I offer some general points in response.

First, although it is true that Matt is indifferent towards his daughter, it is not clear that we need to posit a further entity like an attitude of indifference; one might think that Matt is indifferent towards his daughter simply because he has neither a pro- nor a con-attitude towards his daughter. Second, even if we grant that this new attitude *was* produced, it is not clear that this attitude plays a role in the production of his action. At least, when it comes to how the action was produced, it is unclear what difference the presence of the attitude of indifference makes. Third, let us suppose that such an attitude was produced, *and* that it plays the relevant role in leading to Matt’s decision. We could then modify the case such that, after the attitude of indifference is produced, but before Matt wakes up, the neuroscientists also erase the attitude of indifference. We can now turn to considering bypassing views in further detail.

## Mele’s View

Mele offers two conditions. A sufficient condition for responsibility-level free action, and a necessary condition on direct responsibility intended to rule out cases like that of Paul; though it is not intended to rule out *all* cases of manipulated agents. Mele does not attempt to offer a full analysis of moral responsibility for actions, and neither condition is clearly intended to be exhaustive (Mele, 2019, p. 128). That is, it is consistent with the overall view that there are agents who act freely yet fail to meet the sufficient condition, and that there are manipulated agents who are not directly morally responsible for an action yet meet the necessary condition.

We can begin with the sufficient condition for free action, intended to apply to agents like us. The sense of “free action” in this condition is such that, if an agent freely *A*-s in this sense, and meets freedom-independent conditions on moral responsibility for *A-*ing, then she is responsible for *A*-ing (Mele, 2006, pp. 17, 200):

1b. An agent *A-*s freely if he nondeviantly *A*-s on the basis of a rationally formed deliberative judgment that it would be best to *A*, has no compelled or coercively produced attitudes that influence his deliberative judgment, is well informed on the topic of his deliberation, and is mentally healthy. (Mele, 2006, p. 200)^5^

The historical component of 1b is:

H. The agent “has no compelled or coercively produced attitudes that influence his deliberative judgment.”

Pat, the unmanipulated father, meets H for his decision to take out the loan. Paul, on the other hand, fails to meet H, and thus fails to meet 1b. Mele’s account of what it means for an agent to be compelled to possess an attitude is intricate and only partial, but for attitudes like Paul’s parental values, a part of what accounts for his being compelled to possess them is that the attitude was acquired through bypassing (1995, pp. 171–172). Now consider the case of Matt, who has no compelled or coercively produced attitudes that influence his judgment. After all, he has only lost attitudes, he has not gained any new ones. Matt meets H for his decision, and according to 1b, freely decides.

The specification of B-attitudes suggests a simple revision. Since the set of attitudes an agent is compelled to possess will be a subset of B-attitudes, one simple way to revise H is as follows:

H*: The agent has no B-attitudes or coercively produced attitudes that influence his deliberative judgment

Call the resulting condition 1b*. Although Pat meets 1b* for this decision, Paul and Matt do not, since both of their deliberative judgments were influenced by B-attitudes.

We will return to 1b* later. For now, we can turn to Mele’s necessary condition, intended to yield the verdict that manipulated agents like Paul are not directly responsible for their relevant actions. To say than an agent is directly responsible for an action is to say that they are not responsible for this action merely in virtue of being responsible for some other action (Mele, 2019, p. 11).^6^ Manipulated agents are typically not thought to be *indirectly* responsible for the relevant actions, and for the rest of this paper, one can assume that if a manipulated agent is not directly responsible for an action, then he is not responsible for that action. Mele’s necessary condition is as follows:

- *DMR*. If an agent is directly morally responsible for *A*-ing, then the following is false:
- (1) for years and until manipulators got their hands on him, his system of values was such as to preclude his acquiring even a desire to perform an action of type *A*, much less an intention to perform an action of that type;
- (2) he was morally responsible for having a long-standing system of values with that property;
- (3) by means of very recent manipulation to which he did not consent and for which he is not morally responsible, his system of values was suddenly and radically transformed in such a way as to render *A*-ing attractive to him during *t*; and
- (4) the transformation ensures either
  - (*a*) that although he is able during *t* intentionally to do otherwise than *A* during *t*, the only values that contribute to that ability are products of the very recent manipulation and are radically unlike any of his erased values (in content or in strength) or
  - (*b*) that, owing to his new values, he has at least a Luther-style “inability” during *t* intentionally to do otherwise than *A* during *t*. (Mele, 2019, pp. 127–128)

One concept in need of clarification is that of a Luther-style inability, which appears in 4b. This is a sense of inability used in certain passages from Dennett discussing the phrase famously attributed to Martin Luther: “Here I stand, I can do no other” (Mele, 2019, pp. 62–64). The most concise characterization is expressed by Dennett when he states that: “when I say I cannot do otherwise I mean I cannot because I see so clearly what the situation is and because my rational control faculty is *not* impaired” (Dennett, 1984, p. 133). Notably, this sense of ability is concerned with doing otherwise in relevantly similar circumstances.

The cases of Pat and Paul are intended to be understood as cases of agents who are Luther-style unable to do otherwise than take out the loan (Mele, 2019, pp. 64–65).^7^ How does *DMR* apply to these cases? For Paul’s action of taking out the loan, *DMR* implies that he is not directly responsible (Mele, 2019, p. 67). Pat, on the other hand, meets *DMR* for his similar action, since the conjunction of 1–4 is false with respect to that action.

What does *DMR* have to say about Matt’s action of taking out the loan? Unlike 1b, it does not imply that Matt *is* responsible for his action, since *DMR* is a necessary, not a sufficient, condition for direct responsibility for an action. However, *DMR* also does not imply that Matt is *not* responsible for his action, given that the conjunction of 1–4 is false for his action as well, or so I will argue. Insofar as one wishes to offer a theory that can account for cases like that of Matt as well, one would do well to extend the condition. With this purpose in mind, I suggest provisional revisions to *DMR*.

Conjuncts 1 and 3 help to characterize the degree of change that the victim of manipulation underwent. In order to fit this description, the manipulated agent’s pre-change system of values must have been such as to preclude him from acquiring a desire to perform an action of that type, much less an intention to so act (conjunct 1), and, because of the change due to bypassing, the action must now be attractive to the agent (conjunct 3). Yet Matt’s system of values, prior to the manipulation, was not such as to preclude him from acquiring a desire or intention to take out a loan for a car, nor was it such as to preclude him from performing a selfish action. Consequently, Matt’s system of values did not undergo the sort of change characterized by 1 and 3. His pre-change system of values was, however, such as to preclude him from performing a selfish action *if it conflicted with his parental values*. Thus, I suggest the following (revisions in italics):

(1)* for years and until manipulators got their hands on him, his system of values was such as to preclude *him from deciding, or forming an intention*, to perform an action of type *A in a similar context of practical deliberation*

(3)* by means of very recent manipulation to which he did not consent and for which he is not morally responsible, his system of values was suddenly and radically transformed in such a way as to *not preclude him from deciding, or forming an intention, to perform an action of type* A *in a similar context of practical deliberation*

Both of these claims are true of Matt’s action.^8^ Further, they are also true of Paul’s action, and false of Pat’s, thus avoiding any new problems with those cases.

Conjunct 4, a disjunction, further specifies how significant the manipulation is, in relation to the particular action. Roughly, it states that either, due to the agent’s new values (which are the result of bypassing), he has a Luther-style inability to intentionally do otherwise, or, he is able to intentionally do otherwise at the relevant time, but the only values that contribute to this ability are products of bypassing which are radically different than his erased values. Given that Matt’s selfish values are not new, nor are they the product of bypassing, he fails to meet either disjunct of (4). We can revise 4 as follows:

- (4)* the transformation ensures either
  - (*a*) that although he is able during *t* intentionally to do otherwise than *A* during *t*, the only values that contribute to that ability are, *as a result of the very recent transformation, B-attitudes,* and are radically unlike any of his erased, *or significantly weakened*, values (in content or in strength) or
  - (*b*) that, *owing to his B-attitudes that are a result of the very recent transformation,* he has at least a Luther-style “inability” during *t* intentionally to do otherwise than *A* during *t*.

(4)* is true of Paul, and it is false of Pat; thus, the revision does not create problems with the original cases. If we suppose that Matt lacks the relevant ability, then (4*b*)* is true of him. Suppose instead, that he has the relevant sort of ability in virtue of other attitudes that he retained; suppose, for instance, that he could have instead taken out a loan to install a state-of-the-art pool in his back yard. If this were the case, then these other values would seem to be B-attitudes as well. After all, they would have competed with his parental values in this context of practical deliberation, and the change in their relative strength is due to the erasing of Matt’s parental values. Consequently, Matt would meet (4*a*)* in this scenario. Call this revised view DMR*. This view can now account for the difference between Matt’s and Pat’s actions, as well as the difference between Paul’s and Pat’s actions.

## Positive Views

Mele’s view is sometimes called a *negative historical view,* on which a requirement on responsibility for some action is that she *lacks* a certain sort of history.^9^ On *positive historical views*, in order for an agent to be responsible for an action, she needs to have *had* a certain sort of history.^10^ McKenna (2016)^11^ and Fischer (2012, Chapter 11)^12^ offer positive views. On these views, in order for an agent to be responsible for some action, she needs to have had a certain sort of opportunity to exercise her capacities for control over one’s mental life with respect to, at least some of, the attitudes that led to action. Working out the details of the relevant sort of opportunity will be a complicated matter, and we will return to some of these complications later.

One detail on which the positive views differ concerns which attitudes one needs to have had the relevant opportunity for. Whereas Fischer’s view is concerned with attitudes leading to action in general, McKenna’s view focuses on a specific type of attitude. I mostly focus on McKenna’s view; yet, given that Fischer’s view applies to attitudes in general, the main points I make concerning McKenna’s view will apply to Fischer’s view as well.

McKenna’s view requires that one have had the relevant opportunity for *unsheddable values* that play a role in the production of an action.^13^ Unsheddable values are such that “in normal contexts of practical deliberation, it is not up to an agent during a pertinent duration of time whether or not she possesses that value, nor what degree of strength it has for her” (McKenna, 2016, p. 88). The pertinent duration of time is at the time of action or shortly before it (McKenna, 2016, pp. 88–89). To be clear, the fact that a value is unsheddable *now* does not imply that the value was *always* unsheddable, nor that it always will be.

Using this notion of unsheddable values, McKenna suggests the following view:

*PH*: An agent performs a directly free act and is directly morally responsible for it only if any unsheddable values playing a role in the production of her action arose from a history whereby she was afforded the opportunity to critically assess, endorse, and sustain them from abilities that she possessed, and so none were acquired through means that bypassed those abilities. (McKenna, 2016, p. 97)

One thing to notice with *PH*, as stated, is that if an agent had the relevant opportunity with respect to some unsheddable value, we can infer that it was not acquired through means that bypassed those abilities. This suggests that if it *was* acquired through bypassing, then the agent did not have the relevant opportunity with respect to that value. Thus, the agent needs to have the relevant opportunity *at the time of acquisition.* Even if the agent has the opportunity to assess the value later on, this will not change the fact that she did not have the opportunity relevant to *PH*.

A different version of the view would only require that one have had the relevant opportunity *at some time*, regardless of how the values were acquired. On such a view, bypassing would be relevant insofar as it would preclude the agent’s having the opportunity at the time of acquisition; but if the agent has the opportunity to assess, endorse, and sustain the value *after* acquiring it, she may be responsible for actions that issue from it.^14^

Each of these versions faces its own challenges, and I discuss them separately. We can begin with the first version, on which the agent needs the relevant opportunity at the time of acquisition, reserving “*PH*” for this version of the view.

According to *PH*, Paul is not responsible for his decision, since his unsheddable parental values were acquired via bypassing. Yet Matt’s manipulation did not change any facts about how his selfish values were acquired. If Matt met *PH* for actions that issued from unsheddable selfish values prior to the manipulation, he would seem to meet *PH* for his decision after the manipulation. Did Matt meet *PH* for these pre-manipulation actions?

Notice that *PH* is intended to pick out a relevant difference between agents like Pat and Paul. This means that, at least for some unsheddable values leading to actions, typical agents like Pat need to have had the relevant opportunity at the moment of acquisition. Otherwise, they will fail to meet *PH* as well. If typical agents do not have the relevant opportunity at the time of acquisition, then *PH* fails to capture the difference between typical agents and standard victims of manipulation. If it is possible for typical agents to have this opportunity with respect to some unsheddable values, it is not clear why Matt, a typical agent prior to the manipulation, could not have had it with respect to his selfish values. For our purposes, then, we can assume that Matt meets *PH* for his selfish actions prior to the manipulation; or, at least, that there is a version of the case where he does. Consequently, *PH* fails to account for Matt’s lack of responsibility for his decision.

Making use of the notion of B-attitudes, we can suggest a revision:

PH*: An agent performs a directly free act and is directly morally responsible for it only if, for any unsheddable value, *v*, that plays a role in the production of her action, *v* arose from a history whereby she was afforded the opportunity to critically assess, endorse, and sustain *v* from abilities that she possessed, and *v* is not a B- attitude.

Since Matt’s selfish values were unsheddable, played a role in the production of his decision, *and* were B-attitudes, he fails to meet PH* for his decision. Similarly, Paul does not meet PH* either. On the assumption that Pat met *PH*, he will also meet PH*.

On the second way of understanding McKenna and Fischer’s views, one only needs to have had the relevant opportunity *at some time*, regardless of how the values were acquired. Consider, then, a version of the view along these lines:

*PHO*: An agent performs a directly free act and is directly morally responsible for it only if, for any unsheddable value, *v*, that plays a role in the production of her action, *v* is either not a B-attitude, or if it is, the agent was afforded the opportunity to critically assess, endorse, and sustain *v* from abilities that she possessed.

On this version of the view, one can be directly free and responsible for actions that issue from unsheddable B-attitudes, insofar as one has had the relevant opportunity to asses, endorse, and sustain the attitude at some point or other. This change will not cause problems with the case of Pat, the unmanipulated father.

If Pat met PH*, then he had the relevant opportunity, in which case, he meets *PHO* as well. However, it is not fully clear whether Matt and Paul meet it. This will depend on what it takes to have the relevant opportunity. McKenna suggests that developing this notion will be a complicated matter (McKenna, 2016). For the moment, let us suppose that, whatever the relevant opportunity is, Paul did not have it prior to deciding to take out the loan. If this is true, then it would seem that Matt did not have it either. Matt did not have more time between the manipulation and the decision to assess or endorse his B-attitudes; nor did he have better capacities for control over his mental life. Thus, Matt would seem to fail to meet *PHO* as well.

With these revisions in place, we can now consider how bypassing views compare in a broader context, as well as some potential difficulties that one will face when further developing positive views.

## Further Considerations

We can begin by comparing PH* and *PHO*. To illustrate an advantage held by *PHO*, consider another case. Jim is a talented athlete, and the star player of his high school basketball team. Some basketball players from his rival high school, unhappy with the fact that they have to face him on the court, hatched a plan to get him to play soccer instead. They pooled all of their money together to hire a neuroscientist, but given their limited budget, they can only afford to have the neuroscientist implant a single value, of moderate strength, for playing soccer. When Jim wakes up, he finds that he has a desire to play soccer, and decides to join his friends that afternoon.

He enjoys this game, begins to play frequently, and after a while, decides to quit basketball and join the soccer team instead. Over time, Jim’s value for playing soccer becomes stronger, and after much careful deliberation, he decides to fully commit to soccer. He eventually ends up playing professionally. As Jim’s most recent contract is wrapping up, he faces a choice: he can retire, living off of the substantial fortune he made throughout his career, or he can choose to play for team X, which is known to be owned by, and used as part of the propaganda arm of, a brutal dictatorial government in country Y. Jim decides to sign the contract, and his value for playing soccer, which is unsheddable during the deliberation, plays a role.

In the case of Jim, there are two decisions of interest. First, there is his decision to play soccer with his friends the day after the manipulation. The manipulation in this case is much more modest than that found in the cases of Matt and Paul; and as Mele has suggested, this case would seem to pose a problem for current positive views (Mele, 2019, pp. 54–55).^15^ If one thinks that he is directly responsible for this decision, then one has reason to worry about PH*, and possibly, *PHO*. Nothing in this case would seem to bar the possibility that his value for playing soccer with his friends is unsheddable at the time of deliberation. If it is, then Jim’s decision issues from an unsheddable B-attitude, and thus, according to PH*, Jim is not directly responsible for the decision. *PHO*, on the other hand, may avoid this conclusion, if it turns out that Jim had the relevant opportunity prior to the decision.^16^

Now consider the second decision of interest: Jim’s decision, much later on in his life, to sign the new contract. Most, I presume, would doubt that the origins of the value get Jim off the hook for deciding to sign the contract. However, this value is a B-attitude and unsheddable at the time of deliberation. Consequently, according to PH*, Jim is not directly responsible for the decision. According to *PHO*, on the other hand, Jim can be directly responsible for this decision, since he seems to have had ample opportunity to critically assess, endorse, and sustain the value. Whichever way one develops an account of the relevant opportunity, it is implausible that Jim has not had it prior to deciding to sign the contract. This advantage speaks in favor of *PHO*^17^; however, developing an adequate account of the relevant opportunity will face some challenges, which I mention here.^18^

First, Matt had a similar opportunity with regard to his selfish values prior to the manipulation. Thus, *PHO* needs to be modified in a way that ensures that an agent who meets it had the opportunity *after* its relative strength has been changed via bypassing.^19^ Further, any attempt to develop an account of the relevant opportunity will not only need to meet two further desiderata, it will also need to avoid a potential tension between them.

As mentioned above, in explaining Paul’s lack of direct responsibility for his decision, we can no longer simply appeal to the fact that his action issues from an unsheddable B-attitude. We also need to assess whether he had the relevant opportunity with respect to the attitude at some point after the change. Now recall that, after waking up, Paul is surprised by his new desire to help his daughter. Because of this, he undergoes careful reflection on his parental values, and ends up wholeheartedly embracing them, before deciding to take out the loan. This suggests that Paul both had, and exercised, *some* sort of opportunity to assess, endorse, and sustain his implanted parental values. If *PHO* is to explain why agents like Paul are not responsible for the actions at issue, then the fact that Paul was able to undergo this reflection better not be sufficient for him having had the *relevant* opportunity. A similar complication can arise in the case of Matt, were he to reflect on his selfish values prior to deciding. Call this the *problem of initial reflection*; a desideratum of views like *PHO* is that they avoid this problem. Since agents like Paul can have capacities to assess, endorse, and sustain their values to a similar extent as Pat, the unmanipulated father, the account of the relevant opportunity will need to be somewhat substantial.

A second desideratum, discussed by McKenna, concerns an agent’s first free action. On a view like *PHO*, the agent needs to have had the relevant sort of opportunity with regard to any unsheddable values playing a role in the production of her first free action. Yet as McKenna points out, positive historical views cannot require, for free action, that the agent have performed some free action in the past; this would lead to a problematic regress (McKenna, 2016, pp. 98–9). These views need to be formulated in a way that gives us an account of how an agent can come to perform their first free action, while avoiding this regress. Call this the *problem of first action*. McKenna solves this problem by suggesting that having the relevant opportunity does not require action, but rather “a proper degree of cognitive control or activity” (McKenna, 2016, p. 99), which would seem to block the regress.

A potential tension may arise between the solutions to these two problems. Suppose that, as is often suggested, human agents perform their first free actions and are morally responsible for some of their actions before they become mature adults.^20^ If one’s view is to allow for this, then the proper degree of cognitive control or activity will need to be such that these agents can have it. However, Paul’s initial reflection involved *some* degree of cognitive control or activity, and he can have the capacities to assess, endorse, and sustain his values to a similar extent as unmanipulated Pat. The potential tension between solutions to the problem of first action and the problem of initial reflection arises from the fact that the relevant opportunity will need to be substantial enough to avoid the problem of initial reflection, while thin enough to allow younger agents to have had it by the time they perform their first free action.^21^ This is not to suggest that the tension is irresolvable; this is simply to point out a challenge in developing the account.

On a view like DMR*, some of these problems are avoided. Since it is a negative historical condition, it does not invite the infinite regress that gave rise to the problem of first action. Another difference is that the conditions expressed by DMR* capture a *substantial* change that typical manipulated agents undergo, which helps the view avoid problems with both of Jim’s decisions considered above. Unlike Paul and Matt, Jim’s manipulation produced a fairly minor change, and he seems to meet DMR* for his action of going out to play soccer with his friends the day after the manipulation. Jim’s pre-manipulation system of values, we can suppose, was not such as to preclude him from intentionally performing actions of the same type as going to play soccer with his friends the day after the manipulation. If Jim met DMR* for this action, it would seem that he can also meet it for his action of signing the contract. Further, notice that DMR* only mentions a very recent manipulation or transformation. At the time of his decision to sign the contract, the manipulation was not very recent, giving more reason to think that Jim meets DMR*.

However, there is still work that a notion of opportunity can do in developing a view that incorporates DMR*. As some have suggested, one might think that manipulated agents like Paul and Matt can eventually come to be morally responsible for actions issuing from their problematic attitudes (Cyr, 2020, p. 2390; Fischer & Ravizza, 1998, p. 235; Mele, 2020, pp. 3149–3150).^22^ If this is possible, then it may be in virtue of their having had a certain sort of opportunity to exercise their capacities for control over their mental lives with regard to the problematic attitudes, and how they relate to other, presently or previously held, attitudes. Of course, developing this account will still need to accommodate the problem of initial reflection. Whatever it is that an agent needs to go through in order to regain responsibility for the relevant actions, it will need to involve more than Paul’s initial reflection.

Finally, return to 1b*, the revised version of Mele’s sufficient condition for responsibility-level free action. The suggestion was to revise the historical component of 1b in the following way:

H*: The agent has no B-attitudes or coercively produced attitudes that influence his deliberative judgment.

The case of Jim shows that this revision was too heavy-handed, at least if one wants a sufficient condition which would yield the result that he acted freely the day after the manipulation. Since his deliberation concerning the contract is influenced by a B-attitude, he fails to meet 1b*.^23^ Thus, I suggest a further revision:

H**: The agent meets DMR* and has no coercively produced attitudes that influence his deliberative judgment.

1b**, with H** as a historical component, would tell us that Jim *does* have responsibility-level freedom for his decisions.^24^

## Conclusion

Bypassing views are similar in that, in order to account for the difference between typical agents like Pat and manipulated agents like Paul, they single out attitudes acquired through bypassing. While still focusing on bypassing, this paper has argued that the relevant attitudes to be singled out are rather the broader category of B-attitudes. None of these views, however, suggest that the fact that an action issued from a B-attitude is *sufficient* to undermine direct responsibility for that action.

Bypassing views differ on what further features they incorporate into their account; features picking out other facts of the case that, in combination with the fact that the action issues from attitudes acquired through bypassing, undermine direct responsibly. The positive views further add that the agent has not had a relevant sort of opportunity, and McKenna’s view also limits the view to unsheddable values. Mele’s view, in particular *DMR*, lays out multiple features in its four conjuncts. The revisions suggested in this paper shift the focus to B-attitudes, while accommodating these other features of the original accounts. As suggested, some work still needs to be done, and which challenges one faces will depend on which route one decides to take. One might reasonably wonder, at this point, what implications these revisions have. I offer some brief concluding thoughts in response.

Although discussion of these views tends to revolve around cases involving extreme changes, it is plausible that were the changes to be less extreme, yet still significant, the agent may be *less* responsible for some actions, and for related reasons.^25^ A bypassing view can provide us with a framework for assessing such cases, and the revisions suggested here can help to accommodate a broader set of cases. Further, if we accept the possibility that changes via bypassing can mitigate responsibility for actions, then another question arises: How significant must the change be in order to have *any* effect on an agent’s responsibility for some action? Answering this question will be a complicated matter^26^; but the lower one sets this threshold, the more likely that actual agents undergo such changes.^27^ If actual agents undergo changes of the relevant sort, then the framework that bypassing views provide will be of use for assessing the responsibility of such agents, and these revisions will help to account for a much broader set of cases involving actual agents.

## Footnotes

1 I follow Mele and McKenna in their understanding of “*S* values *X*”: “*S* at least *thinly values X* at a time if and only if at that time *S* both has a positive motivational attitude toward *X* and believes *X* to be good” (Mele, 1995, p. 116) (See also (McKenna, 2016, p. 88)).

2 These sorts of cases are often employed against compatibilism—the view that moral responsibility is compatible with the truth of determinism—and often involve the additional feature that the agents are in a deterministic universe. I omit discussion of this part of the debate. At least for cases that are the focus of this paper, there are similarly problematic variations set in indeterministic settings (Cyr, 2016; Haji & Cuypers, 2001; King, 2013; McKenna, 2016, p. 88; Mele, 2019, pp. 124–126; Tognazzini, 2014).

3 There is another set of views that do not appeal to bypassing in order to explain why agents like Paul are not free and/or responsible. Rather, they appeal to the presence of the manipulator’s intention (Barnes, 2015; Yaffe, 2003), or to the causal source of the agent’s action, which in these cases, is the manipulator’s intentional action (Deery & Nahmias, 2017). Further, Waller (2014) suggests that the effective intention is relevant to the degree of responsibility. This paper, however, will just focus on bypassing views.

4 Importantly, this is consistent with the agent’s retaining these attitudes after the change.

5 Mele also offers a sufficient condition that an incompatibilist can accept. The historical component of this view is identical to that in 1b (2006, p. 201).

6 An example sometimes used to contrast direct with indirect responsibility is that of a driver who, given his intoxicated state, does not have control over his action of hitting a pedestrian. If he is responsible for hitting the pedestrian, it is likely in virtue of a previous choice he made over which he had control.

7 *DMR* is intended to be about overt actions (Mele, 2019, p. 68). Because of this, I talk about the action of taking out the loan, rather than the decision to do so.

8 Conjunct 2 refers to a property specified in 1. Since Matt’s pre-change system of values did not have such a property, he failed to meet 2 as well. Since 1* specifies a property that Matt’s pre-change system *did* have, he meets 2 on the revised view, and there is no need to revise that conjunct.

9 Mele adopts a negative view in order to leave open the possibility of instant agents who are responsible for their first actions. Instant agents are full-fledged agents who come into being just moments before acting (Mele, 2019, Chapter 3).

10 A further view, which I do not discuss in this paper, is Haji and Cuypers’s view (2008). Their view is a hybrid; it is negative for the first actions that an agent is responsible for, and positive for later actions.

11 Although McKenna develops a bypassing view, he does not endorse it.

12 Fischer is one of the initial proponents of historical views of responsibility (Fischer 1994, Chapter 8; Fischer and Ravizza 1998, Chapters 7–8). Here I discuss the most recent development of the view.

13 This term was introduced by Mele (1995, p. 153). Mele and McKenna seem to use the term in different ways, and Mele has opted to continue without it (2019, p. 68). In this paper, “unsheddable” will be used in McKenna’s sense.

14 This sort of view might be suggested by McKenna’s claim that “PH only requires that a free agent have been afforded an opportunity to critically assess, endorse, and sustain relevant values from abilities that she possessed” (2016, p. 100), or by Fischer’s claim that “[w]hat is crucial is that we have a fair and reasonable opportunity to filter new elements of our mental economy through our character as a whole” (2012, p. 198).

15 Jim’s decision to play soccer with his friends is similar to Mele’s case of Carl, which he employs against an earlier version of McKenna’s view (McKenna, 2012). However, the criticism applies to the positive historical views under consideration in this paper as well.

16 In responding to a different case, McKenna suggests that manipulation cases involving only a small change will not have much dialectical force (2017, pp. 579–80), since it is plausible that victims of this minor manipulation may still be morally responsible for the relevant action. It is not clear, however, what part of *PH* would capture this.

17 It is worthwhile to note that this advantage may turn out to be fairly significant. Depending on what it takes to engage the capacities for control over one’s mental life, it may turn out that many of our values were changed, or even acquired, in a way that did not engage them. If this is right, then, as mentioned above, *PH* fails with regard to the cases of Paul and Pat.

18 For discussion of further complications in developing this account of opportunity, see (McKenna, 2016, pp. 98–101).

19 One further consideration to keep in mind when doing so is the possibility of multiple changes via bypassing over time. If Jim is manipulated *again*, after he signs the contract, and this affects the relative strength of his value for playing soccer, then we might need to modify *PHO* to ensure that he has had the opportunity after the most recent such change.

20 I intend to remain vague on when exactly this happens; perhaps it happens during adolescence for most agents, perhaps it happens before then. It may be worth noting, as others have, that agents performing their first free actions may be free and responsible to a lesser extent than typical adult agents. For some discussion of what are sometimes called “little agents” performing their first free actions, in the context of discussion of historical views, see Cyr (2020), McKenna (2021) and Mele (2021).

21 In a recent paper, Taylor Cyr presents a related challenge, focusing on constitutive luck, and intended to apply to all bypassing views (2020). My challenge is aimed specifically at positive historical views, since only these invite the worry of an infinite regress. See Mele (2020) for a direct response to Cyr’s challenge, and Haji and Cuypers (2008, pp. 58–60) and Mele (1995, pp. 79–84; 2006, pp. 79–84) for responses to some of Cyr’s concerns.

22 Mele gives a story in which this sort of thing happens (2020, pp. 3149–3150), though he does not give an account of it. Discussion of this story may be of some use for producing a general account. Yet, there is at least some reason to think that the story might face problems with the case of Matt. In the story Mele gives, the manipulated agent comes to autonomously possess the implanted values, at least in part, by evaluating them in light of other autonomously held values that she had prior to the manipulation. Yet Matt’s initial reflection after the manipulation is done in light of such values as well.

23 It is also noteworthy that Jim meets the original 1b.

24 However, 1b** may still have room for improvement. Though I do not develop this concern here, I point to a possible case. Consider an agent who meets *DMR**, but only because the manipulation was not very recent (since it was not very recent, 3* is not true of this agent). For instance, suppose that Paul falls into a coma right after the manipulation, and wakes up a couple of months later. Depending on how the case is fleshed out, we might worry that 1b** would tell us that he is responsible for actions that issue from the implanted parental values, when he is, in fact, not.

25 Or, if one prefers, less blameworthy/praiseworthy; or lesser kinds or amounts of blame/praise may be fitting, deserved, or appropriate. I do not wish to take a stand on how best to understand claims about more or less responsibility, though see Coates (2019) for a discussion.

26 As will determining which features affect the degree of significance. Some candidates: the number of attitudes changed, the degree of change in individual attitudes, the centrality of these attitudes to the person’s “deep” self, whether the changed attitudes are first- or second-order, and how resistible the changed attitudes are.

27 There already are, albeit rare, cases that might involve significant enough changes for bypassing views, as they stand, to tell us something about the agents’ responsibility (Burns and Swerdlow, 2003; De Marco, 2019; Sharp & Wasserman, 2016). Of particular interest for the revisions suggested here may be the use of anti-libidinal drugs to reduce the libido of certain sex offenders; for discussion of their use and legality in various jurisdictions, see Forsberg (2018).
